# Vitiligo and Alopecia Areata After Donor Lymphocyte Infusions in a Child With Relapsed Acute Myeloid Leukemia

**DOI:** 10.7759/cureus.52810

**Published:** 2024-01-23

**Authors:** Fan-Yu Liao, Yi-Lun Wang, Yu-Chuan Wen, Chia-Chi Chiu, Tsung-Yen Chang, Tang-Her Jaing

**Affiliations:** 1 Department of Nursing, Chang Gung Memorial Hospital, Taoyuan, TWN; 2 Department of Pediatrics, Division of Hematology/Oncology, Chang Gung Memorial Hospital, Taoyuan, TWN

**Keywords:** acute graft vs host disease, acute myeloid leukemia (aml), donor lymphocyte infusion, alopecia areata, vitiligo

## Abstract

Rarely do patients with chronic graft-versus-host disease (cGVHD) experience vitiligo and alopecia areata. Nevertheless, the exact cause of vitiligo and alopecia areata is still not fully understood. The patient experienced a relapse of acute myeloid leukemia (AML) following a second complete remission after undergoing HLA-6/8 mismatched unrelated donor hematopoietic cell transplantation (HCT). Achieving full donor chimerism was successful during the initial stages of the transplant. Nevertheless, the molecular evidence of measurable residual disease remained, prompting the administration of donor lymphocyte infusions (DLI) following a dose-escalation protocol. After three cycles of DLI given at two-month intervals, the circulating blasts eventually vanished. After the third DLI dose, vitiligo developed despite achieving molecular remission. The dermatologist confirmed the presence of vitiligo and alopecia areata, along with cutaneous cGVHD. The outcome was the complete elimination of the molecular presence, and the patient experienced both clinical and molecular remission for a period of five years following DLI. Based on our observations, it was found that DLI could effectively eradicate molecular leukemia in cases of AML relapse after HCT. The development of vitiligo and alopecia areata was influenced by the destruction of melanocytes due to autoimmune reactions caused by cGVHD.

## Introduction

Vitiligo and alopecia areata are not frequently observed in individuals who have undergone allogeneic hematopoietic cell transplantation (HCT). It has been established that there is a connection between vitiligo and chronic graft-versus-host disease (cGVHD) [1-3]. Although it has been observed in a limited number of patients after HCT and is believed to be caused by T-cell deregulation [4-6], there have been few reports of its occurrence after donor lymphocyte infusions (DLI) in the English literature [7,8]. Thus, the exact causes of the correlations are still unclear. This report details a case of notable hypopigmentation, which is consistent with vitiligo, that occurred in a patient with acute myeloid leukemia (AML) after undergoing HCT.

## Case presentation

In January 2018, our institution received a referral for transplant evaluation of an 11-year-old boy with AML who was in the second remission status. The patient received a transplantation of unmanipulated peripheral blood stem cells from a 6/8 human leukocyte antigen-matched, unrelated male donor in May 2018. There was no record of depigmentary dermatoses in either the patient or the donor. His parents had no skin diseases. The conditioning for HCT involved the use of busulfan and cyclophosphamide. The GVHD prophylaxis regimen involved the administration of methotrexate and cyclosporine A one year after HCT.

**Figure 1:**
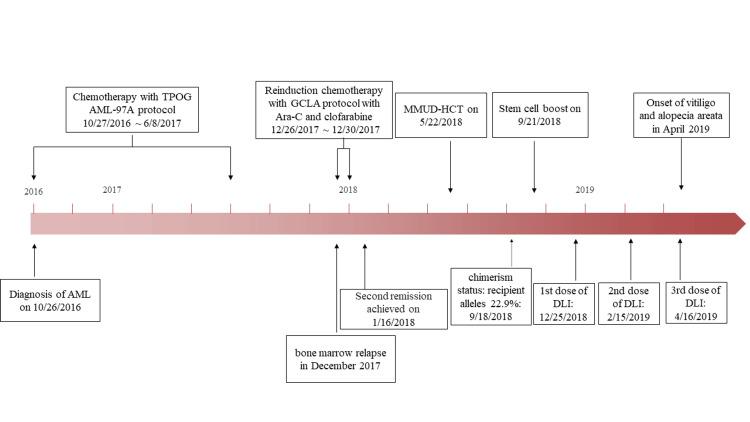
An overview of the clinical timeframe for the development of vitiligo and acute myeloid leukemia Abbreviation: AML: acute myeloid leukemia; DLI: donor lymphocyte infusion; GCLA: Gemcitabine, Cisplatin, and L-Asparaginase; MMUD-HCT: mismatched unrelated donor hematopoietic cell transplantation; TPOG: Taiwan Pediatric Oncology Group

On the other hand, a decline in donor chimerism after four months post-transplant is often seen as an indication of graft failure or relapse. The administration of reinduction chemotherapy was subsequently accompanied by an increase in the dosage of donor stem cells to attain clinical remission. He then underwent three cycles of preemptive DLI, which were given in increasing doses every two months (Figure 1).

**Figure 2:**
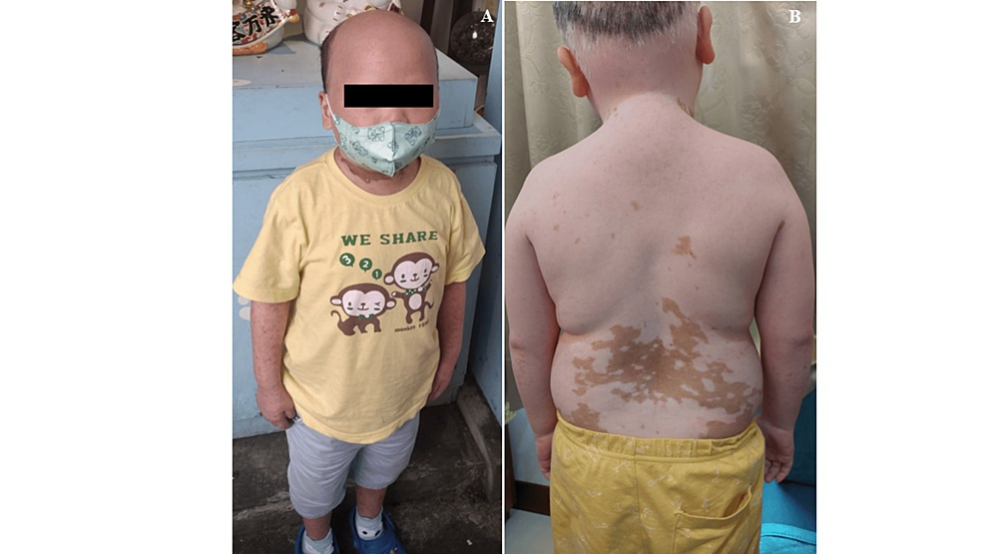
Hyperpigmentation that is spotty or patchy, followed by depigmentation that is prevalent throughout the body

After four months of DLI, hair loss occurred in the frontal and temporal areas, resembling non-scarring and ophiasis-like patterns. On examination, the patient presented with depigmented macules on the face, neck, trunk, and extremities, which were consistent with vitiligo. The areas showed depigmentation of body hair. The clinical finding in Figure 2A was indicative of a vitiligo diagnosis. Furthermore, he experienced alopecia areata (Figure 2B). The lesions treated with topical steroids showed no signs of improvement. The depigmentation did not show any signs of spreading during his follow-up, and as of January 2024, he had remained free of leukemia for five years.

## Discussion

The risk of sensitization to minor histocompatibility antigens in the blood donor, which can lead to a higher risk of GVHD, is increased by excessive pretreatment and blood transfusion before HCT [9]. DLI has proven effective in achieving remission in cases of AML relapses following HCT. Nevertheless, there is limited documentation on the complete elimination of its molecular presence. Additionally, the destruction of melanocytes may be enhanced by infusing more lymphocytes after DLI [8]. Mussetti et al. [10] found that an elevated CD3+ graft content was linked to a higher incidence of all-grade cGVHD.

DLI is an immunotherapy that boosts the body's natural defense against leukemia, leading to long-lasting remission [11]. This treatment has the potential to eliminate measurable residual disease and provide a chance for long-lasting remission in certain patients [12]. This phenomenon has both positive and negative aspects. The GVL effect plays a crucial role in eliminating leukemic cells. The correlation between the DLI dose and GVHD or the GVL effect was not always consistent. It is important to note that the GVL effects and GVHD are distinct phenomena [13].

The cutaneous manifestations of cGVHD exhibit a wide range of variability and can resemble established autoimmune diseases such as systemic sclerosis and Sjögren syndrome [14]. However, the characterization of vitiligo and alopecia areata in cGVHD has been limited [15]. There is evidence to suggest that GVHD, particularly cGVHD, may contribute to the development of autoimmune-mediated vitiligo.

## Conclusions

The etiology of vitiligo and alopecia following DLI can be elucidated as follows: Initially, it is possible that cGVHD has elicited an immune response that selectively targets melanocytes in the skin. Additionally, the introduction of a greater quantity of lymphocytes following DLI can potentially play a role in the eradication of melanocytes. Finally, the transmission of vitiligo and alopecia from the donor to the recipient following HCT might result in the persistent occurrence of vitiligo and alopecia in the long run. There is a noticeable lack of reports on vitiligo and alopecia areata in cGVHD studies, with most of the available studies being limited to case studies.

## References

[1] Nguyen J, Singh N, Afifi S, Giralt S, Lacouture ME, Busam KJ, Hassoun H (2020). Vitiligo following autologous hematopoietic stem cell transplantation. Clin Lymphoma Myeloma Leuk.

[2] Joge RR, Kathane PU, Joshi SH (2022). Vitiligo: a narrative review. Cureus.

[3] Čeović R, Desnica L, Pulanić D (2016). High frequency of cutaneous manifestations including vitiligo and alopecia areata in a prospective cohort of patients with chronic graft-vs-host disease. Croat Med J.

[4] Zuo RC, Naik HB, Steinberg SM (2015). Risk factors and characterization of vitiligo and alopecia areata in patients with chronic graft-vs-host disease. JAMA Dermatol.

[5] Wang Y, Li S, Li C (2019). Perspectives of new advances in the pathogenesis of vitiligo: from oxidative stress to autoimmunity. Med Sci Monit.

[6] Dai J, Hight RS, Grullon K, Xiao TL, Stein SL (2023). Vitiligo-like manifestations of graft-versus-host disease in a pediatric population. Pediatr Dermatol.

[7] Totani A, Amin H, Bacchi S, Lewis I (2020). Vitiligo following stem-cell transplant. Bone Marrow Transplant.

[8] Li Z, Rubinstein SM, Thota R (2016). Immune-mediated complications after hematopoietic stem cell transplantation. Biol Blood Marrow Transplant.

[9] Koyama M, Mukhopadhyay P, Schuster IS (2019). MHC class II antigen presentation by the intestinal epithelium initiates graft-versus-host disease and is influenced by the microbiota. Immunity.

[10] Mussetti A, De Philippis C, Carniti C (2018). CD3+ graft cell count influence on chronic GVHD in haploidentical allogeneic transplantation using post-transplant cyclophosphamide. Bone Marrow Transplant.

[11] Ye Y, Yang L, Yuan X, Huang H, Luo Y (2021). Optimization of donor lymphocyte infusion for AML relapse after allo-HCT in the era of new drugs and cell engineering. Front Oncol.

[12] Dholaria B, Savani BN, Labopin M (2020). Clinical applications of donor lymphocyte infusion from an HLA-haploidentical donor: consensus recommendations from the Acute Leukemia Working Party of the EBMT. Haematologica.

[13] Krieger E, Toor AA (2020). Can graft vs. leukemia effect be uncoupled from graft vs. host disease? An examination of proportions. Front Immunol.

[14] Hill GR, Betts BC, Tkachev V, Kean LS, Blazar BR (2021). Current concepts and advances in graft-versus-host disease Immunology. Annu Rev Immunol.

[15] Wang Y, Hu W, Lin F, Xu AE (2023). Generalized vitiligo after stem cell transplantation: a case report. Clin Cosmet Investig Dermatol.

